# The Establishment of Asian Black Bear (*Ursus thibetanus*) Fibroblast Cell Line

**DOI:** 10.3390/ani16030436

**Published:** 2026-01-30

**Authors:** Yujuan Hu, Wanrong Song, Liwei Zhang, Longyue Yuan, Yipeng Jin

**Affiliations:** College of Veterinary Medicine, China Agricultural University, Beijing 100193, China

**Keywords:** *Ursus thibetanus*, skin fibroblasts, cellular immortalization, hTERT

## Abstract

The Asian black bear (*Ursus thibetanus*, UT), a Class II protected species in China, presents significant challenges for dermatological research due to limited availability of skin specimens and technical difficulties in fibroblast isolation. To address this, this study developed an immortalized fibroblast cell line through lentiviral-mediated introduction of human telomerase reverse transcriptase (hTERT). The hTERT gene fragment was amplified via PCR and cloned into a eukaryotic expression plasmid using homologous recombination. The recombinant plasmid was packaged into lentiviral particles using a three-plasmid system (psPAX2, pMD2.G) for stable transduction of primary *Ursus thibetanus* skin fibroblasts (UTSF). Puromycin selection enabled isolation of positive clones, which were subsequently expanded to establish an immortalized cell line. Comprehensive validation through Western blotting, PCR and immunofluorescence confirmed successful genomic integration and sustained expression of hTERT. This study successfully immortalized the skin fibroblasts of black bears and established a fibroblast cell line of Asian black bears. This immortalized UTSF cell line provides a critical in vitro model system for investigating cutaneous physiology and pathogen interactions in this protected species.

## Simple Summary

The Asian bear (*Ursus thibetanus*, UT) is a a protected species, and research into its skin physiology and diseases is severely limited by the scarcity of tissue samples and the difficulty of maintaining cells in the laboratory. To overcome these challenges, this study established a permanent (immortalized) line of skin fibroblast cells from the Asian black bear. By introducing the human hTERT gene into primary skin cells, we enabled them to divide indefinitely while retaining their normal genetic structure and biological functions. We further validated that these cells can effectively simulate wound healing processes, such as cell migration, under inflammatory conditions. This newly established cell line provides a sustainable and valuable tool for studying skin health and diseases in Asian black bears, supporting conservation efforts without the need for repeated sampling from wild animals.

## 1. Introduction

Cell immortalization refers to the process of cell culture in vitro that, due to their own genetic changes or external stimuli, can be long-term subculture and proliferation [1]. Spontaneous immortalization occurs at exceptionally low frequencies, with reported rates ranging from 10^−5^ to 10^−6^ in rodent cells and less than 10^−12^ in human cells [2]. To overcome this biological constraint, researchers employ genetic engineering strategies to introduce exogenous immortalizing agents into target cells through techniques like gene transfection to increase the rate of immortalization and establish immortalized cell strains to endow in vitro cultured cells with unlimited proliferation capacity and no intercellular differences. Currently, common methods for cell immortalization include hTERT, SV40LT, Epstein–Barr virus, human papilloma virus, oncogenes and mutant tumor suppressor genes.

Telomeres are specialized DNA–protein complexes at eukaryotic chromosomes ends. They have a simple, repetitive, highly conserved DNA sequence rich in guanine (G) bases [3]. Their primary biological functions are preventing chromosomal DNA degradation, terminal fusion deletions and abnormal recombination. By safeguarding chromosome ends, they avert chromosomal fusion and the instability of extensive genetic information, thereby preventing cell division cessation and the onset of cellular senescence. Telomere length is related to mitotic division number. In vitro-cultured cells lose 50 to 200 bp of telomere length per division [4]. In normal animal cells, telomeres progressively shorten as cells divide. When critically short, they cannot protect chromosome ends, leading to fusion and genomic instability. This causes cells to cease proliferation and ultimately undergo apoptosis. Telomere length reflects a cell’s replication history and potential, called the “mitotic clock”. In primary cultured cells, the stable expression of the TERT gene can maintain telomere length and facilitate cellular immortalization [5].

Therefore, the mechanism of telomerase-induced immortalization mainly involves enhancing telomerase activity for cellular immortalization. Telomerase, a specialized reverse transcriptase with an intrinsic RNA template and telomerase reverse transcriptase (TERT), uses its RNA as a template to extend telomeres from the 3′-OH terminus of telomeric DNA or synthesize new telomeric DNA, countering telomere shortening from replicative senescence. This maintains telomere length, preventing senescence and apoptosis. In normal cells, telomerase has minimal activity, but it is significantly higher in immortalized cells. For example, introducing hTERT into retinal epithelial cells activates endogenous telomerase and extends replicative lifespan [6]. Further studies show TERT, as the catalytic subunit of telomerase, enhances its activity, delays senescence and promotes immortalization. Cells transduced with hTERT keep their original structure, function and biological characteristics [7]. Thus, hTERT transduction is one of the most widely used methods for cellular immortalization.

Skin fibroblasts in the reticular dermis secrete and deposit integrins and extracellular matrix (ECM) components like collagen, elastic fibers and binding proteins in connective tissues. Due to their ECM-secretion capacity, they play key roles in various cutaneous repair processes, including skin aging, photoaging [8,9], inflammation, burn or mechanical injuries [10], scar formation [11] and tumor-related pathologies.

Skin inflammation promotes the migration of dermal fibroblasts. The cytoskeleton, particularly microfilaments, is recognized as a critical regulator of cellular motility. Studies have demonstrated that Stathmin mediates TNF-α-induced fibroblast migration by facilitating pseudopodium formation. Cytochalasin B, a well-established pharmacological agent for microfilament research, effectively suppresses various microfilament-dependent cellular movements. The establishment of permanent cell lines is a vital strategy for species where primary tissue availability is limited, such as the *Ursus thibetanus*, a Class II protected species in China. By providing a renewable and standardized in vitro platform, this approach circumvents the logistical and ethical constraints associated with repeated primary sampling in wildlife research.

## 2. Materials and Methods

### 2.1. Skin Tissue Collection, Primary Fibroblast Isolation and Cell Culture

The skin tissue sample was obtained from a deceased wild *Ursus thibetanus*, approximately 3 years old, during a necropsy conducted by authorized personnel in a nature reserve. The animal was not sacrificed for this study and the sample collection complied with relevant wildlife protection regulations in China. Ethical approval for the use of animal-derived materials was obtained from the Animal Welfare and Ethical Review Board of China Agricultural University (Approval No. AW30301202-3-6).

The *Ursus thibetanus* skin specimens were disinfected with 75% ethanol, depilated and aseptically dissected into approximately 2 cm × 2 cm tissue blocks. Following three sequential washes in calcium/magnesium-containing phenol red-free Hank’s balanced salt mixture (Solarbio, Beijing, China) supplemented with 1% penicillin–streptomycin (Gibco, Waltham, MA, USA), the tissue blocks were minced into 1 mm^3^ fragments. These fragments were transferred to culture dishes and enzymatically digested with 0.25% Trypsin-EDTA (Gibco, Waltham, MA, USA) under continuous agitation in a 37 °C shaker for 1 h to ensure optimal tissue dissociation. Terminate the digestion by adding the culture medium and rinse the tissue blocks repeatedly with Hank’s. These fragments were transferred to culture dishes and enzymatically digested with 0.1% collagenase type Ⅰ (1 mg/mL) (Gibco, Waltham, MA, USA) in PBS under continuous agitation in a 37 °C shaker for 4 h to ensure optimal tissue dissociation. The resultant cell suspension was filtered through a 40 µm cell strainer and subsequently centrifuged at 1000 rpm for 10 min. After supernatant removal, the pellet was resuspended in PBS and recentrifuged under identical parameters. Following final resuspension in complete medium, the cells were plated in 10 cm culture dishes and maintained at 37 °C with 5% CO_2_, with daily monitoring of cell growth status. Upon reaching 80–90% confluence, primary cells were triple-rinsed with PBS and treated with 2 mL of 0.25% Trypsin-EDTA (Gibco, Waltham, MA, USA) at room temperature for 3 min. Complete medium was added in equal volume to neutralize enzymatic activity, followed by gentle pipetting to detach rounded dermal fibroblasts. The cell suspension was centrifuged at 1000 rpm for 5 min, after which the trypsin-containing supernatant was aspirated. The cells were resuspended in fresh complete medium and subcultured under standard culture conditions.

### 2.2. Determination of the Minimum Lethal Concentration of Puromycin in UTSF

**Figure 1:**
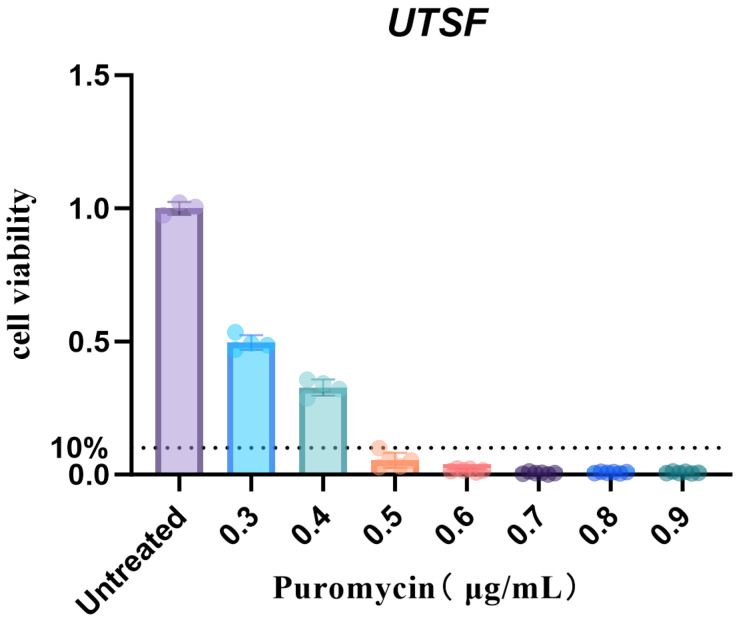
Determination of the minimum lethal concentration of puromycin in UTSF. As can be seen from Figure 1, when the concentration of puromycin is 0.5 µg/mL, the killing rate of cells is greater than 90%. Therefore, 0.5 µg/mL is finally selected as the minimum killing concentration. (The dashed line indicates the threshold at which the cell mortality rate reaches 90%).

Puromycin (MCE, Junction, NJ, USA) was diluted in complete medium at concentrations ranging from 0.3 to 0.9 μg/mL, with a gradient interval of 0.1 μg/mL. Fibroblasts were seeded into 96-well plates at a density of 1 × 10^4^ cells per well (designated as Day 0, D0). Starting from D1, the original medium was replaced with puromycin-supplemented medium corresponding to each concentration. The puromycin-containing medium was refreshed every 48 h. On Day 10, cell viability was assessed using the CCK-8 assay (*n* = 6) and data were analyzed with GraphPad Prism 10.1.2. The minimum lethal concentration (MLC) of puromycin was determined to be 0.5 μg/mL (Figure 1).

### 2.3. Construction of the Plasmid

The hTERT gene was amplified from the pCMV-EGFP-TERT (human)-Neo plasmid using primer pairs:

pCMV-EGFP-TERT(human)-Neo-F: GATCTATTTCCGGTGAATTCATGCCGCGCGCTCCCC;

pCMV-EGFP-TERT(human)-Neo-R: GCTCTAGAACTAGTCTCGAGTCAGTCCAGGATGGTCTTGAAGT.

Subsequently, primers with specific homologous arms were employed to construct the PLVX-hTERT-Puro sequence from the PLVX-IRES-Puro backbone:

PLVX-hTERT-Puro-F: GATCTATTTCCGGTGAATTCATGCCGCGCGCTCCCCGC;

PLVX-hTERT-Puro-R: GCTCTAGAACTAGTCTCGAGTCAGTCCAGGATGGTCTTGAAGT.

**Figure 2:**
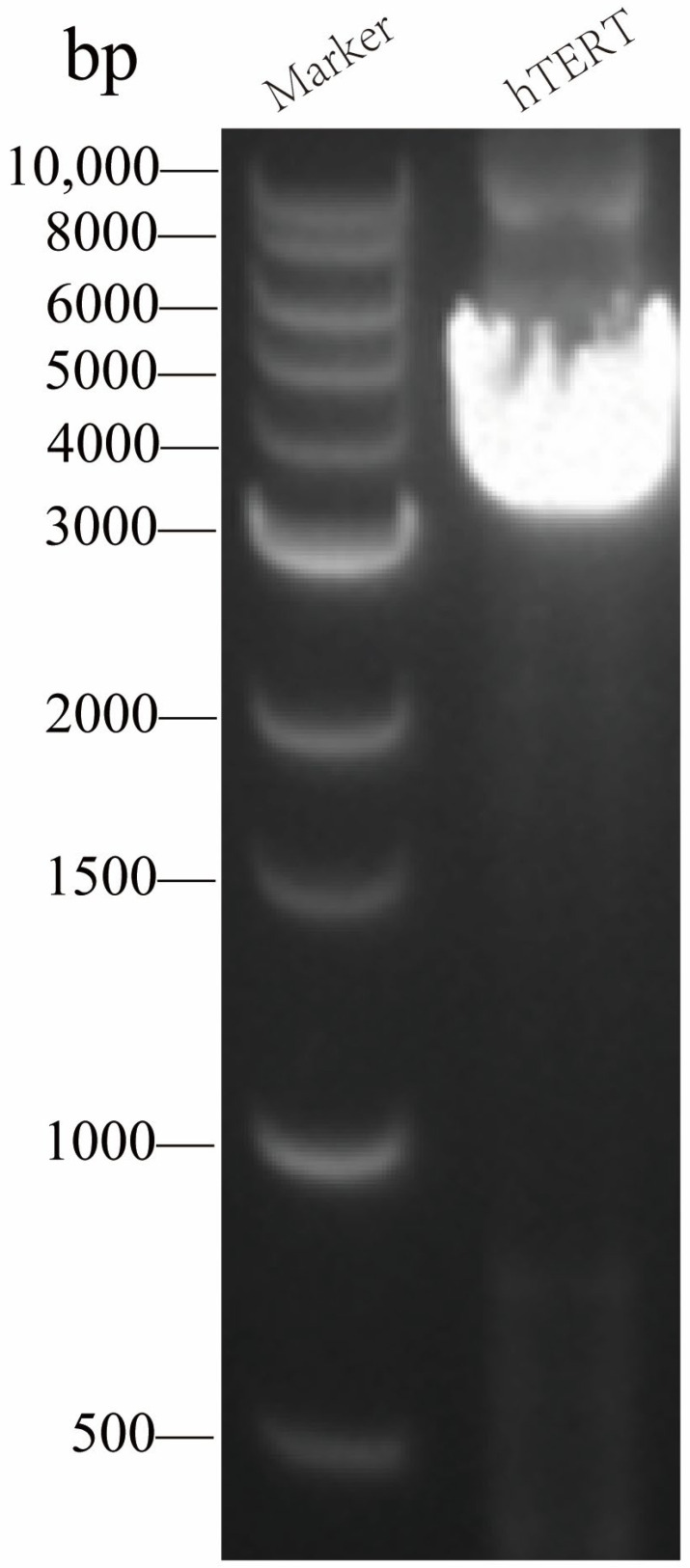
The amplification result of the hTERT gene by PCR of the PLVX-hTERT-Puro plasmid. Successfully obtained the hTERT gene from the pCMV-EGFP-TERT (human)-Neo plasmid.

Following PCR amplification of the hTERT gene fragment with homologous arm extensions, the amplified hTERT fragment containing homologous arms and the restriction-digested eukaryotic expression plasmid (PLVX-IRES-Puro) were assembled using the Uniclone One Step Seamless Cloning Kit (Genesand, Beijing, China) to generate the lentiviral packaging shuttle plasmid PLVX-hTERT-Puro (Figure 2). The constructed plasmid was subsequently confirmed by sequencing.

### 2.4. Lentiviral Packaging and Infection

Healthy HEK 293T cells were resuscitated and subcultured at least twice, ensuring that the cell density reached approximately 80% the following day after passage. Two hours prior to transfection, the medium was replaced with fresh complete culture medium (without antibiotics). A lentiviral package was prepared by mixing plasmids in the ratio of PLVX-hTERT-Puro: psPAX2: pMD2.G = 3:2:1, totaling 24 µg of plasmid DNA. To this, 60 µL of Lipofectamine 2000 was added for transfection. The 24 µg of DNA was diluted in 1.5 mL of Opti-MEM™ reduced serum medium (Gibco, Waltham, MA, USA) and mixed gently. For the Lipofectamine™ 2000 transfection reagent (Thermo Fisher Scientific, Waltham, MA, USA), 60 µL was diluted in 1.5 mL of reduced serum medium, incubating at room temperature for 5 min. The Lipofectamine mixture was slowly added to the plasmid solution, mixed gently and incubated at room temperature for 20 min. Then, the resulting complex was added dropwise to the cell culture medium, gently mixing by rocking the plate back and forth, and the cells were incubated at 37 °C in a CO_2_ incubator. After 4 h, the medium was replaced with fresh complete culture medium. The cell supernatant containing the lentivirus was collected 48 h after the media change and stored at 4 °C. Culturing of the cells continued with an additional 10 mL of complete medium and, after 24 h, the cell supernatant was collected again.

Concentration of Lentivirus: The collected supernatant is filtered through a 0.22 µm cell filter. For every 30 mL of the filtered initial virus solution, 7.5 mL of 5X PEG-8000 NaCl stock solution (virus solution: concentrated solution = 4:1) was added. This was incubated overnight on a shaker at 4 °C. It was then centrifuged at 4000× *g* for 30 min at 4 °C, the supernatant removed, and the lentivirus pellet resuspended in DMEM. This was stored at −80 °C and used as needed.

### 2.5. The Transfection of UTSF with Lentivirus

The skin fibroblasts with good growth status were seeded in 6-well plates and cultured in a 37 °C, 5% CO_2_ incubator. When the cell confluence reached over 80%, the cells were washed three times with PBS. Then, 1 mL of culture medium containing 4 μg/mL Polybrene (Beyotime, Haimen, China) was added, followed by the addition of concentrated lentivirus, with a blank control well (no lentivirus added). After 4 h of incubation at 37 °C, 5% CO_2_, 1 mL of culture medium containing 4 μg/mL Polybrene was added. After 24 h, the medium was replaced with fresh, Polybrene-free complete medium and cultured for another 24 h. The medium was then replaced with medium containing 0.5 μg/mL puromycin and cultured continuously. The puromycin-containing medium was changed every 48 h until the cells in the blank control wells were completely dead.

### 2.6. Western Blot (WB)

hTERT-UTSF cells were seeded in a 6-well plate and allowed to grow until they reached approximately 90% confluence. The cells were washed three times with PBS and then lysed using radio immunoprecipitation assay buffer (RIPA) supplemented with Phenylmethanesulfonyl fluoride (PMSF). The lysate was collected into 1.5 mL centrifuge tubes and incubated on ice for 30 min. The samples were then centrifuged at 4 °C at 1000× *g* for 30 min and the supernatant containing the proteins was collected. The protein concentration was measured using the Bicinchoninic Acid Assay (BCA) and adjusted to the desired concentration with RIPA buffer. An equal volume of 5× loading buffer was added and the samples were denatured at 100 °C for 5 min. Equal amounts of protein samples were loaded for SDS-polyacrylamide gel electrophoresis (SDS-PAGE) and then transferred to a nitrocellulose membrane. The membrane was blocked with 5% non-fat dry milk in TBST for 30 min, followed by three washes with 0.05% Tris-buffered Saline with Tween20 (TBST) for 5 min. The membrane was incubated overnight on a shaking incubator at 4 °C with the primary antibody. After three washes with 0.05% TBST for 5 min, the membrane was incubated with the goat anti-rabbit secondary antibody at room temperature on a shaking incubator for 45 min. Finally, after three washes with 0.05% TBST, the antigen–antibody complexes were exposed using chemiluminescent reagent. The results were observed and recorded using a chemiluminescent image analysis system.

### 2.7. Polymerase Chain Reaction (PCR)

The puromycin-resistant clonal cells derived from lentivirus-transfected hTERT-UTSF were subjected to RNA extraction using Trizol reagent (Thermo Fisher Scientific, Waltham, MA, USA), followed by cDNA synthesis through reverse transcription. PCR amplification was subsequently performed to verify the successful transduction of the hTERT plasmid into the cellular genome. Specific primers targeting the hTERT plasmid sequence were designed using Primer Premier 5 software, yielding an 883 bp amplification product. The primer sequences were as follows:

hTERT-F: 5′-GCCGCCTGAGCTGTACTTTGTC-3′;

hTERT-R: 5′-CGTCTGGAGGCTGTTCACCTG-3′.

### 2.8. Indirect Immunofluorescence Assay (IFA)

hTERT-UTSF were cultured on cell climbing slides in a 24-well plate until they reached approximately 70% confluence. The culture medium was aspirated and the cells were washed three times with ice-cold PBS. Subsequently, 4% paraformaldehyde was added for overnight fixation at 4 °C. After aspirating the paraformaldehyde, the cells were permeabilized with 0.5% Triton X-100 (Beyotime, Haimen, China) at room temperature for 10 min, followed by washing three times with ice-cold PBS. The slides were then blocked with Fetal Bovine Serum (FBS) at room temperature for 30 min before overnight incubation with the primary antibody. After three additional washes with ice-cold PBS, the slides were incubated with a fluorescence-labeled secondary antibody at room temperature in the dark for 45 min. Following another three washes with ice-cold PBS, the cells were stained with dilactate (DAPI) for 10 min. Finally, mounting medium containing anti-fade agent was applied, allowed to dry and observations were made using an Olympus BX51^TM^ (Nikon, Tokyo, Japan) fluorescence microscope with 40× objective lens and the images were merged in the FUJI ImageJ (Windows, 2024).

Meanwhile, immunofluorescence staining of vimentin was performed on hTERT-UTSF. The operation steps were the same as those mentioned above, and the primary antibody was changed to vimentin antibody.

### 2.9. Chromosome Analysis

When the P20 hTERT-UTSF are in the logarithmic growth phase, colchicine (Beyotime, Haimen, China) is added to the cell culture medium to achieve a final concentration of 0.2 µg/mL and the mixture is gently mixed. After continuing incubation at 37 °C for 4 h, the cells are washed three times with PBS and treated with 0.25% Trypsin-EDTA for digestion. Following digestion, the cells are centrifuged to obtain a cell pellet, which is then resuspended in 8 mL of 0.075M KCl (VivaCell Biosciences, Shanghai, China), mixed thoroughly and subjected to hypotonic treatment in a 37 °C water bath for 30 min. Subsequently, 1 mL of Carnoy’s fixative (V methanol: V glacial acetic acid = 3:1) is added and mixed for pre-fixation. The cells are then centrifuged at 1500 rpm for 10 min, and the supernatant is discarded. An additional 8 mL of Carnoy’s fixative is added, mixed and left at room temperature for 15 min. The centrifugation process is repeated at the same conditions. After discarding the supernatant, an appropriate amount of fixative is added to create a cell suspension. Slides are pre-treated in a −80 °C freezer, positioning one end of the slides higher to create a 15° angle. The cell suspension is dropped from a distance of 30 cm above the cold slide (before the ice film disappears) to allow for natural dispersion. The slides are then placed in an oven to dry for 1–3 h and stained with Giemsa Stain (Beyotime, Haimen, China) for 15 min. After washing with purified water, the slides are air-dried at room temperature and observed under an oil immersion microscope.

### 2.10. In Vitro Inflammation Modeling

hTERT-UTSF were cultured in DMEM supplemented with 20% FBS and antibiotics under standard conditions (37 °C, 5% CO_2_). After 48 h of incubation, cells were passaged at 80% confluency. Subsequent experiments were conducted using cells in the logarithmic growth phase. To simulate early inflammatory conditions in vitro, cells were treated with 20 ng/mL recombinant TNF-α protein for 24 h.

## 3. Results

### 3.1. Characterization of UTSF

**Figure 3:**
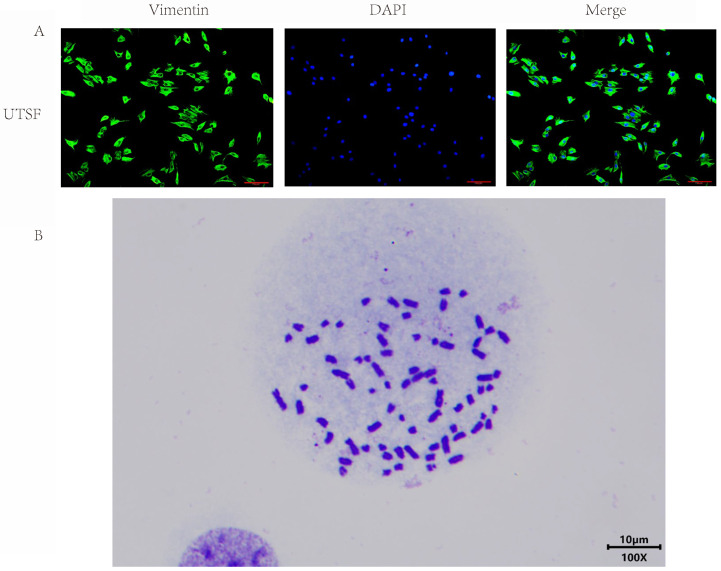
Characterization of UTSF. (**A**) Vimentin identification of UTSF in the P10 by immunofluorescence. Vimentin (green); nuclei (blue). (**B**) Karyotype identification of hTERT-UTSF in the P20. Scale bar = 10 µm.

As can be seen from Figure 3A, UTSF has obvious fibroblast-specific protein vimentin signal. Combined with the isolation method of UTSF and the analysis of cell morphology, it is determined that the isolated cells are fibroblasts from the skin of *Ursus thibetanus*. The karyotype analysis was conducted on the P20 hTERT-UTSF and the cell division phase was observed in Figure 3B. The number of hTERT-UTSF chromosomes obtained is 74, which is consistent with the chromosome number of *Ursus thibetanus* [12,13]. This indicates that the cells obtained in this experiment are cells from *Ursus thibetanus*. At the same time, it also indicates that the ectopic expression of hTERT can extend the lifespan of primary UTSF without changing the chromosomes and successfully obtained immortalized hTERT-UTSF.

### 3.2. The hTERT Gene Exhibits Stable Expression in hTERT-UTSF

**Figure 4:**
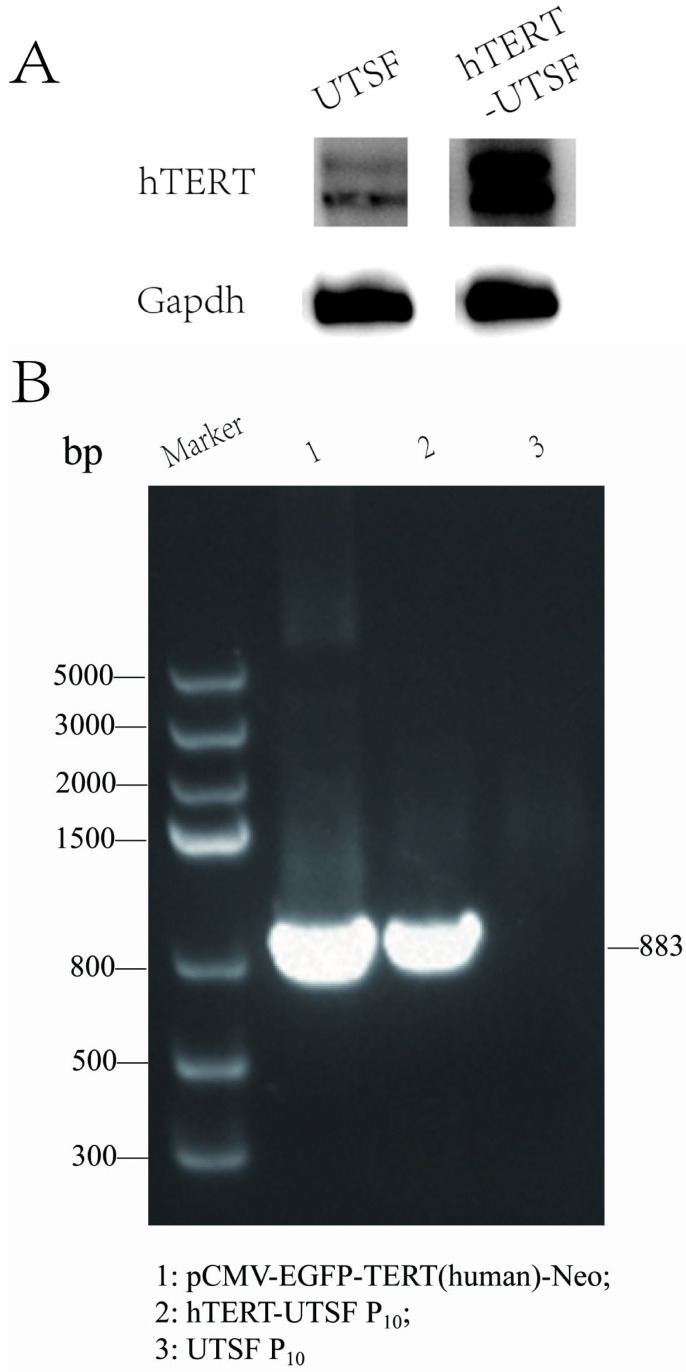
The expression of hTERT in hTERT-UTSF. (**A**) Detect the expression of hTERT using Western blot method. (**B**) Detect the expression of the hTERT gene in P10 hTERT-UTSF using PCR technology.

**Figure 5:**
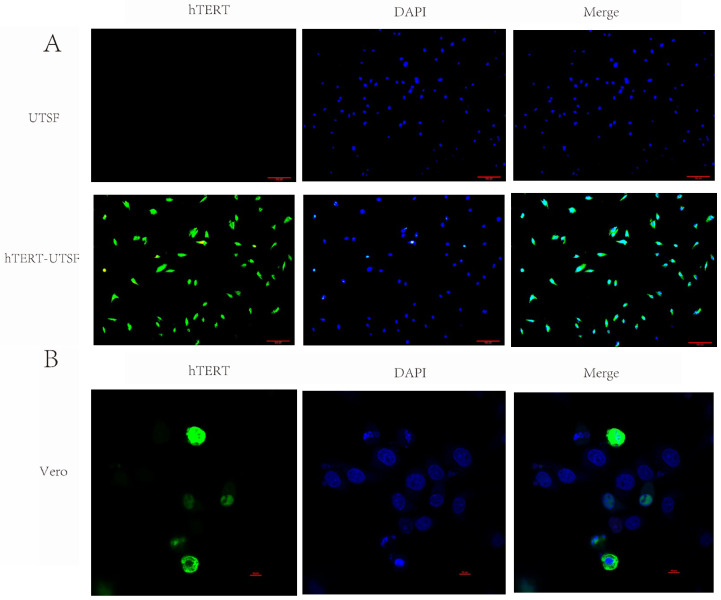
Observation of the subcellular localization of hTERT in hTERT-UTSF. (**A**) Collect primary UTSF and hTERT-UTSF samples from 24-well plates, fix them with IFA and observe. hTERT (green); nucleus (blue). Scale bar = 50 µm. (**B**) Subcellular localization of the hTERT gene. Transfect Vero cells with pCMV-EGFP-TERT(human)-Neo plasmid. After 24 h of transfection, observe the fluorescence images using confocal fluorescence microscopy. hTERT (green); nuclei (blue).

Using the pCMV-EGFP-TERT(human)-Neo plasmid as the positive control, PCR amplification was performed on the passage 10 (P10) hTERT-UTSF genome. The results showed that using the DNA of hTERT-UTSF as the template could amplify a specific fragment of 883 bp, which was consistent with the size of the amplified fragment of the positive control (Figure 4A). Western blot analysis revealed that the expression level of hTERT in hTERT-UTSF was significantly higher than that in the primary UTSF (Figure 4B). These results indicate that hTERT is stably expressed as an exogenous gene in hTERT-UTSF, suggesting that the hTERT gene has been integrated into the UTSF genome through the lentivirus packaging system. After transfection of pCMV-EGFP-TERT(human)-Neo plasmid into Vero cells for 24 h, fluorescence images were observed and taken using confocal fluorescence microscopy. It could be seen that hTERT was located in the cell nucleus and bound to the cell chromosomes by using the Nikon A1HD25^TM^ (Nikon, Tokoyo, Japan) confocal microscope with 100× objective lens and the images are merged in the NIS-Elements Viewer (Figure 5B).

**Figure 6:**
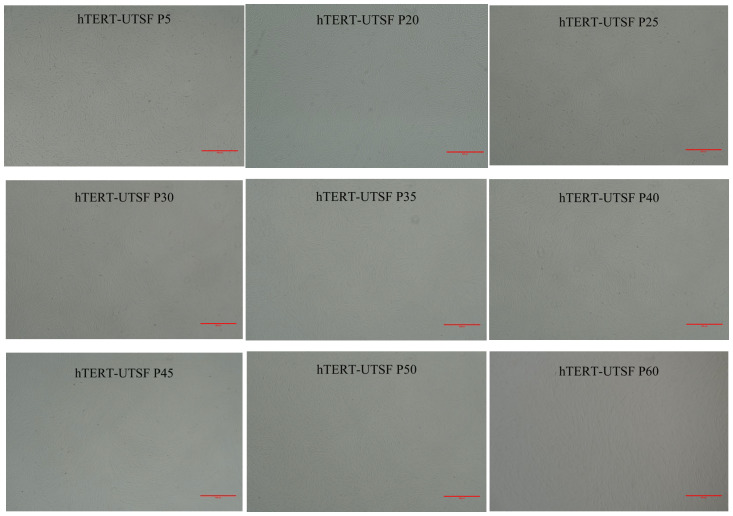
Morphological observation of hTERT-UTSF growing cells. Microscopic images of hTERT-UTSF from different passages were captured. Scale bar = 500 µm.

**Figure 7:**
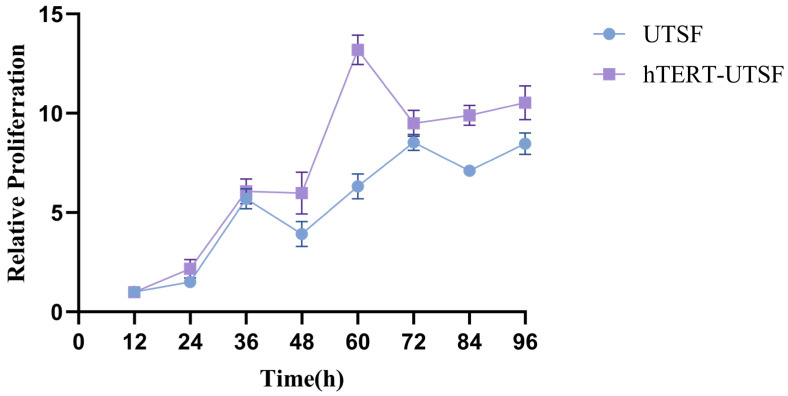
Cell growth curves of UTSF and hTERT-UTSF. Primary UTSF and hTERT-UTSF were seeded in 96-well plates at a density of 5 × 10^4^ cells per well. At 12 h, 24 h, 36 h, 48 h, 60 h, 72 h, 84 h and 96 h, the cell growth curves were determined by CCK-8 assay. The cell proliferation at 96 h was detected and the cell growth curve was plotted based on the absorbance value at 96 h.

As shown in Figure 6 the subcloned hTERT-UTSF maintained a uniform fibrous morphology at P5, P20, P25, P30, P35, P40, P45, P50 and P60, indicating that hTERT-UTSF supports uniformity characteristics during subculture. Compared with primary UTSF, hTERT-UTSF exhibited higher viability upon thawing and a faster recovery rate. The growth curve is shown in Figure 7.

### 3.3. Effects of the Inflammatory Environment on hTERT-UTSF

The hTERT-UTSF represents an in vitro skin wound healing model that can be utilized to study wound repair in black bears. In both wild and captive settings, black bears frequently sustain skin injuries due to territorial or mating conflicts, which pose threats to their health. As a Class II protected species in China, such injuries not only impact individual well-being but also present challenges for population conservation efforts. Currently, the scarcity of accessible skin tissue samples and the lack of suitable cell lines derived from black bear skin significantly hinder in-depth research on the mechanisms underlying their wound healing processes.

**Figure 8:**
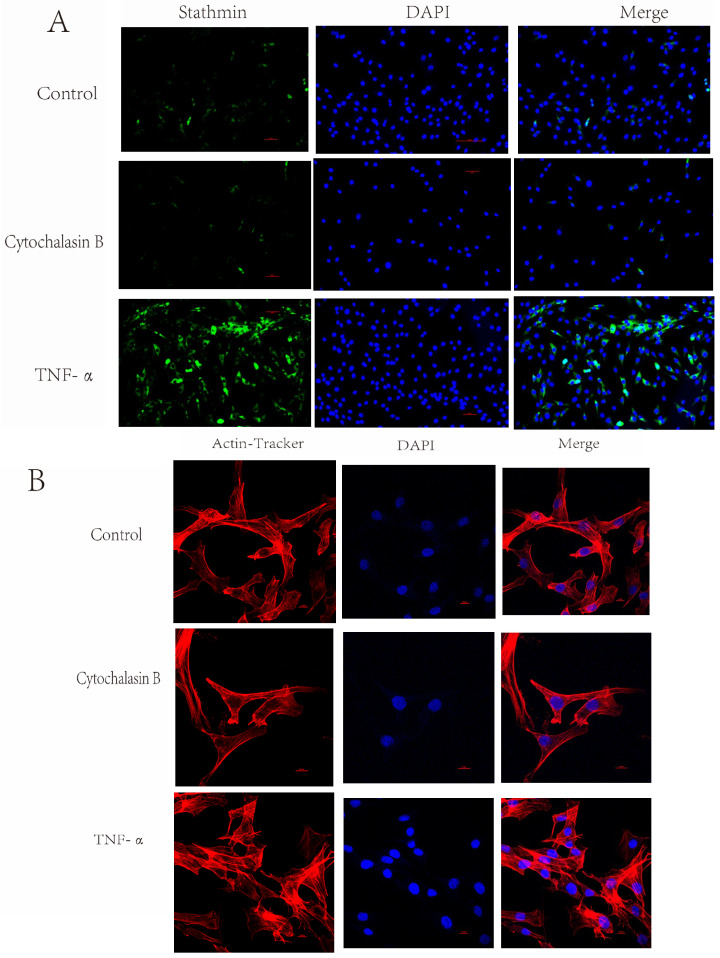
Effects of TNF-α on pseudopodia formation in hTERT-UTSF. (**A**) Immunofluorescence staining of Stathmin expression in hTERT-UTSF cells treated with TNF-α and cytochalasin B. Nuclei were counterstained with DAPI. Scale bar = 50 µm. (**B**) TNF-α treatment promotes filopodia formation in hTERT-UTSF. Fibroblasts were untreated (Control), treated with TNF-α, or pre-treated with cytochalasin (an actin polymerization inhibitor) followed by TNF-α. Actin filaments were stained with rhodamine-phalloidin (red). TNF-α-treated fibroblasts exhibited a significant increase in filopodia formation compared to the control. This effect was abolished by cytochalasin B pre-treatment, confirming the actin-dependent nature of filopodia. Nuclei were counterstained with DAPI. Scale bar = 50 µm.

This study successfully established the hTERT-UTSF cell line for the first time by introducing the hTERT gene via a lentiviral packaging system. To evaluate the migratory capacity of hTERT-UTSF during wound healing, the study conducted migration assays under conditions simulating an early inflammatory environment. As shown in Figure 8A, pseudopod formation was observed in inflammatory conditions, suggesting enhanced cellular motility. Furthermore, scratch wound assays and immunofluorescence staining demonstrated stronger migratory ability under inflammatory conditions and confirmed pseudopod formation and migration in hTERT-UTSF cells. These findings indicate that hTERT-UTSF can serve as a valuable cellular model for further investigation into skin wound healing mechanisms.

## 4. Discussion

Cellular immortalization has become a hot topic in biomedical research in recent years [14]. The hTERT gene is currently widely used in the establishment of transgenic animal models and the immortalization of various human and animal cells [14,15,16,17,18,19]. After a few divisions, cells enter the senescence (M1) phase. They do not die and continue to divide into the M2 phase, with chromosomes shortened to the limit [20,21,22,23,24,25,26,27]. The ectopic introduction of the hTERT gene, which complexes with endogenous telomerase RNA to reconstitute telomerase activity, effectively enables cells to bypass replicative senescence and achieve immortalization [28]. In current research, lentiviral vectors and liposome transfection are predominantly used. The lentivirus-mediated ectopic expression of hTERT has been proven to immortalize human skin keratinocytes [29]. Compared to other published hTERT-immortalized fibroblast lines, such as those derived from the dermal fibroblasts [29,30,31] and fetal bovine skin fibroblast lines [18], the hTERT-UTSF line maintained its typical spindle-shaped fibroblast morphology and stable growth kinetics up to 60 passages. For instance, similar to hTERT-immortalized human dermal fibroblasts, the hTERT-UTSF line bypasses the “Hayflick limit” without exhibiting the drastic phenotypic drift often observed in primary cells. They have strong proliferative capacity and stable expression of immortalization traits. Finally, hTERT-UTSF clonal cells with both immortalized and original characteristics were obtained and identified, providing materials for in vitro studies of *Ursus thibetanus* skin-related diseases.

Furthermore, some earlier studies utilized viral oncogenes like the SV40LT for immortalization. In contrast to fibroblasts immortalized with the SV40LT antigen, which are prone to accumulating chromosomal rearrangements and genomic instability [32], hTERT-immortalized fibroblasts consistently retain their original species-specific karyotype [33]. In contrast, the comparison with hTERT-only models in other large mammals reveals that hTERT maintains a stable, species-specific diploid karyotype, which is critical for the reliability of in vitro toxicological and physiological assays. By aligning the findings with these diverse models, the researchers confirm that the hTERT-UTSF line serves as a high-fidelity surrogate for primary Asian black bear fibroblasts, bridging the gap between limited clinical samples, the need for large-scale and reproducible experimental data.

## 5. Conclusions

This study successfully established an immortalized *Ursus thibetanus* skin fibroblast cell line (hTERT-UTSF) through lentiviral-mediated transduction of the hTERT gene. Comprehensive validation, including genomic integration analysis, consistent protein expression and long-term proliferation assays, demonstrates that the hTERT-UTSF line successfully bypasses replicative senescence while consistently maintaining the original species-specific karyotype and fundamental functional properties. Compared to primary cells, this immortalized line offers a stable, consistent and renewable cellular resource. This achievement not only overcomes the technical and ethical constraints associated with obtaining primary skin specimens from this protected species but also provides a robust and standardized in vitro platform for future research in Asian black bear dermatology, physiology and conservation medicine.

## Data Availability

All data generated or analyzed during this study are included in this published article.
